# Assessment of Spatio-Temporal Variation and Driving Mechanism of Ecological Environment Quality in the Arid Regions of Central Asia, Xinjiang

**DOI:** 10.3390/ijerph18137111

**Published:** 2021-07-02

**Authors:** Xu Bi, Bianrong Chang, Fen Hou, Zihan Yang, Qi Fu, Bo Li

**Affiliations:** 1 College of Resources and Environment, Shanxi University of Finance and Economics, Taiyuan 030006, China; geo_bnu@163.com (X.B.); houyuhoufeng@163.com (F.H.); 2 Faculty of Geographical Science, School of Natural Resources, Beijing Normal University, Beijing 100875, China; 201631190022@mail.bnu.edu.cn; 3 College of Humanities, Tianjin Agricultural University, Tianjin 300384, China; changbianrong1220@163.com; 4 School of Politics and Public Administration, Soochow University, Suzhou 215123, China; 5 Collaborative Innovation Center for New Urbanization and Social Governance in Jiangsu Province, Soochow University, Suzhou 215123, China; 6 Center for Chinese Urbanization Studies of Soochow University, Suzhou 215123, China

**Keywords:** ecological quality, mountain–basin system, MODIS data products, spatio-temporal changes, driving mechanism

## Abstract

Grassland ecosystems are increasingly threatened by pressures from climate change and intensified human activity, especially in the arid region of Central Asia. A comprehensive understanding of the ecological environment changes is crucial for humans to implement environmental protection measures to adapt to climate change and alleviate the contradiction between humans and land. In this study, fractional vegetation coverage (FVC), leaf area index (LAI), gross primary productivity of vegetation (GPP), land surface temperature (LST), and wetness (WET) were retrieved from Moderate-Resolution Imaging Spectroradiometer (MODIS) satellite remote sensing products in 2008 and 2018. Principal component analysis (PCA) was used to establish the MODIS data-based ecological index (MODEI) in the study area, and the spatial differentiation characteristics and driving mechanism of ecological quality in the last ten years were explored. The results showed that: (1) FVC, GPP, LAI, and WET had positive effects on the ecological environment, while LST had a negative impact on the ecological environment. FVC and GPP were more significant than other indicators. (2) The MODEI showed a spatial pattern of “excellent in the north and poor in the south” and changed from north to south in the study area. (3) From 2008 to 2018, the average MODEI of Fuyun County increased from 0.292 to 0.303, indicating that the ecological quality in Fuyun County became better overall. The improved areas were mainly located in the summer pastures at higher elevations. In comparison, the deteriorated areas were concentrated in the spring and autumn pastures and winter pastures at lower elevations. The areas where the ecological environment had obviously improved and degraded were distributed along the banks of the Irtysh River and the Ulungur River. (4) With the increase in precipitation and the decrease in grazing pressure, the MODEI of summer pasture was improved. The deterioration of ecological environment quality in spring and autumn pastures and winter pastures was related to the excessive grazing pressure. The more significant changes in the MODEI on both sides of the river were associated with implementing the herdsmen settlement project. On the one hand, the implementation of newly settled villages increased the area of construction land on both sides of the river, which led to the deterioration of ecological quality; on the other hand, due to the increase in cropland land and the planting of artificial grasses along the river, the ecological quality was improved. The study offers significant information for managers to make more targeted ecological restoration efforts in ecologically fragile areas.

## 1. Introduction

The arid area of China is located in the hinterland of the Eurasian continent, on the northern edge of the Qinghai–Tibet Plateau. The specific geographical location has created unique landforms, vegetation, and soil types in arid areas of China, and is the critical protection area of ecologically fragile sites in China. The alternate distribution of mountains and basins is a fundamental feature of the physical geography of the arid regions in northwestern (NW) China. Zhang defined this combination of terrestrial ecosystems as a mountain–basin system (MBS) from ecology [1].

The zone system is connected by energy flow, water flow, life flow, value flow, and culture flow. It is the most essential and precious natural resource and mode of action in the arid zone and thus becomes the supporting system of natural ecology and human society in the region. The ecosystem is fragile due to the particularity of the geographical location and geomorphic structure [2]. The MBS in arid regions is ecologically sensitive, with significant ecological and environmental problems such as vegetation degradation and difficulty in restoration. In recent years, with the vigorous advancement of the construction of national ecological civilization, the ecology of some areas has been highly valued, especially in areas with fragile environments. The resource and environmental carrying capacity assessment and ecological quality comprehensive assessment have become hot issues nationwide in recent years. Therefore, the timely and rapid monitoring of ecological environment changes in ecologically vulnerable areas has become the focus of researchers and relevant government departments.

At present, remote sensing technology is widely used in ecological environment research due to its advantages of speed and use in real time. It has become an essential means of ecological environment assessment [3,4]. In recent years, remote sensing technology has been successfully applied to ecological monitoring, particularly for the evaluation of ecosystems including forests [5], grasslands [6], cities [7], and river basins [8,9]. It can make up for the deficiency of traditional semi-quantitative ecological evaluation methods and provide an effective research method for regional ecological evaluation, and thus many scholars have studied ecological environment monitoring and assessment based on remote sensing methods [10]. In the selection of an ecological environment remote sensing monitoring index, some studies evaluated the ecological environment by a single index, such as using the net primary productivity (NPP) index to evaluate grassland ecology [11], using the land surface temperature (LST) to assess urban heat islands [12,13,14], using green vegetation fraction (GVF) to estimate the ecological status and its changes in the riparian zone [15], or using impervious surface coverage to evaluate the urban ecological environment [16]. Although a single index is easy to understand and easy to calculate, the ecological environment is a complex system in the existing ecosystem, and the ecological environmental quality is controlled by multiple ecological factors [17], leading to difficulties in characterizing the quality of the ecological environment by only a single ecological factor [18]. Therefore, it is necessary to comprehensively evaluate the ecological environment from the perspective of the integrity of the ecosystem and explore the change rules of each ecological factor and its synergistic relationship to establish a comprehensive index that can couple multiple factors [19].

The other type is the comprehensive evaluation model constituted by the fusion of remote sensing data and other types of data, such as the ecological environment index (EI) proposed in the Technical Specifications for Ecological Environmental Status Assessment (HJ/T192-2006) issued by the Ministry of Ecology and Environment of the People’s Republic of China. The index is widely used in the field of ecological evaluation [20,21], and it includes remote sensing data (vegetation cover index, water network density index, biological abundance index), land surface monitoring data (land degradation index), and annual statistics (environmental quality index). However, statistical data are generally obtained through annual statistical data at the county level and above. Therefore, it is difficult to reflect the spatial differentiation of ecological quality by the ecological quality index based on the county level. In addition, the accuracy of information extraction for land use is low in areas with complex topography. In general, the application of the EI has great limitations.

Liu et al. [22] extracted the vegetation coverage, biological abundance, and land degradation index based on MODIS data and built an evaluation model to analyze the ecological environment changes in Shaanxi Province. Xu [23] constructed the remote sensing ecological index (RSEI) model through the vegetation index, humidity component, land surface temperature, and dryness index, which was used for monitoring the changes in ecological environmental quality and has been widely used [24,25,26]. The RSEI is an ecological index based on remote sensing information, which is the most intuitive reflection of natural factors and the ecological environment by integrating multiple indicators as the leading indicator. The index is composed entirely of remote sensing data, so it has the characteristics of timeliness and rapidity. The index realizes the objective and quantitative evaluation of the regional ecological environment and can invert the ecological environment quality in different regions well [27,28,29,30].

At present, most studies have focused on the eastern coastal areas or the central areas where human activities are more frequent, with few reports on the ecologically fragile areas in the arid area of NW China. Therefore, the studies on evaluating ecological quality based on satellite products in the arid region of NW China promote the interdisciplinary integration of remote sensing and ecology, and enrich the theory and method system of ecological evaluation.

The fragile ecological environment in NW China is highly sensitive to global climate change [31,32]. Fuyun County is located in the northernmost part of the Xinjiang Uygur Autonomous Region, which is a typical MBS area, and a crucial fragile ecological protection area of the country. In recent years, grassland desertification, biodiversity reduction, carbon sink loss, and other phenomena have appeared in this region [33], which not only endangered the production, life, and development of local farmers and herders, but also threatened regional ecological security [34]. Currently, studies on the MBS in the arid region of NW China mainly focus on grassland productivity change [35], land use change [36], ecosystem services [37], and household livelihood change [38]. However, few studies have focused on monitoring and evaluating the ecological quality of the MBS in the arid region.

As an essential part of the ecosystem, vegetation plays various roles, such as climate regulation, water conservation, and habitat provision. Especially in the arid region of NW China, vegetation has a more significant and vital impact on the ecological environment, while data of animal and microbial factors are lacking and difficult to quantify. Therefore, this study adopted the vegetation as the evaluation object, with the help of remote sensing products, selected the vegetation growth and the main non-biological factors that affected its growth as the evaluation indicators, and established the evaluation model to comprehensively evaluate the ecological environment of the vegetation in the MBS in NW China.

The improved RSEI [39] is introduced in this paper. Fractional vegetation cover (FVC), gross primary productivity (GPP), leaf area index (LAI), land surface temperature (LST), and wetness (WET) were used to represent ecological environment greenness, total vegetation productivity, vegetation quality, regional heat, and humidity, and a MODIS-based ecological environment quality index (MODEI) was created. Therefore, this study aimed to: (1) calculate the ecological environment indicators (FVC, GPP, LAI, LST, and WET) using MODIS data in 2008 and 2018; (2) construct the MODEI using the principal component analysis (PCA) method and analyze the spatial and temporal variation characteristics of the MODEI in Fuyun County, and discuss the spatial differentiation characteristics of the MODEI; (3) combine climate change, land use change, grazing pressure, and other factors to analyze the reasons for the changes and explore the possible driving mechanism. This research can provide a new objective evaluation perspective for the ecological quality evaluation of MBSs in arid areas and help enrich the theory and method system of ecological evaluation. In addition, the results of this study can provide a scientific basis for the effective management and sustainable use of grassland in MBSs in arid regions.

## 2. Materials and Methods

### 2.1. Study Site

**Figure 1:**
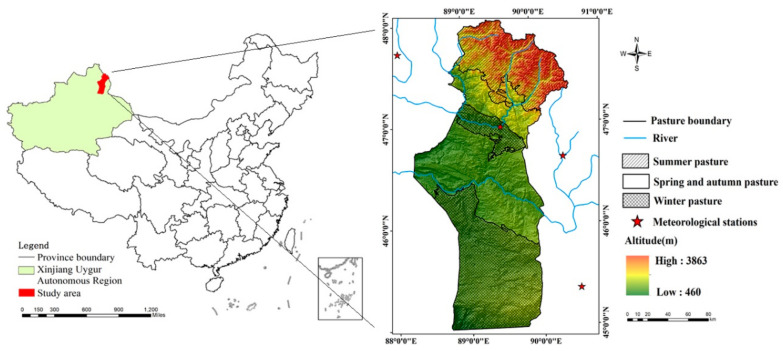
The geographical map of the location of the study area.

Fuyun County is located in the eastern part of Altay (45°00′–48°03′ N, 88°10′–90°31′ E), the northernmost part of Xinjiang Uygur Autonomous Region, with an area of approximately 32,186 km^2^. It stretches from the southern base of the Altai Mountains to the north of the Junggar Basin, with elevation descending from 3863 m to 460 m (Figure 1). It is a typical representative of an MBS in Central Asia, and the high elevation gradient creates a distinct vertical zonal climate and vegetation type. Fuyun County is located in the temperate arid and semi-arid climate zone, with an average temperature of 3.0 °C, annual precipitation of 189.6 mm, and annual evaporation of 1970 mm. The Irtysh River and the Ulungur River are two major rivers in the study area.

Grassland is the primary ecosystem type in this region, covering almost 80% of the whole area. Herders graze on the grasslands at different altitudes in different seasons. According to the time that the herdsmen utilize the grassland, the grasslands are divided into different seasons (summer pasture, spring and autumn pasture, and winter pasture). Summer pastures are mostly high-quality pastures and are mainly distributed in the Altai Mountains above 1200 m. Grazing starts in early June and ends in early September for about 90 days. Winter pastures are distributed below 500 m, and most of them are inferior pastures, which are mainly composed of temperate deserts. The grazing period starts in early December and ends in late March of the following year for about 120 days.

Spring and autumn pastures are distributed between winter and summer pastures, mainly in hilly and plain desert areas with an altitude of 500 m to 1200 m. The grazing time is divided into two seasons: spring grazing starts in late March and ends in early June, and the utilization time is about 70 days; autumn grazing starts in early and late September and ends in early December with a utilization time of 85 days.

### 2.2. Data Sources

The MODIS (NASA, Washington, United States) installed on Terra and Aqua satellites has a scan width of 2330 km and acquires global observation data every 1 to 2 days. Due to its wide coverage and short return visit period, it is widely used in large-scale ecological research. All indicators are derived from EOS/MODIS remote sensing products (https://lpdaacsvc.cr.usgs.gov/appeears/ (accessed on 5 March 2021)), and the data of MODIS products are shown in Table 1.

The above MODIS data products have undergone radiometric calibration and atmospheric correction, and the data products were acquired from July to September in 2008 and 2018. This period is the vegetation growing season, and it is easy to distinguish between vegetation areas and bare snow areas. In this paper, MODIS Reprojection Tool software was used for data mosaic and projection conversion of each indicator raster datapoint. The unified resolution was 500 m × 500 m, and the projection was GCS_WGS_1984. In the process of data screening, the data with less cloud cover and of high quality were selected to calculate the average value of the five indicators from July to September. Water area, bare land, and snow area cannot participate in ecological evaluation effectively because some indicators have no value, so the water area, bare land, and snow area were excluded by mask. The meteorological data used in this study were obtained from the China Meteorological Science Data Sharing Service Network (http://data.cma.cn/ (accessed on 5 March 2021)), and the temperature and precipitation data from 54 national meteorological stations in Xinjiang Uygur Autonomous Region were collected. The statistical data were obtained from the Statistical Yearbook of Fuyun County.

### 2.3. Methods

#### 2.3.1. Calculation of Indicators

Vegetation is the most important component of the ecosystem. Based on former studies [28,39], this study selected five indicators related to vegetation to directly reflect the quality of the ecological environment [7]. Among them, GPP is denotes the physiological characteristics and growth status of vegetation, and the LAI reflects the growth quality of vegetation further. Based on the normalized difference vegetation index (NDVI), FVC was further extracted to represent vegetation growth status and coverage degree, and FVC is an important index to measure regional greenness directly. LST and WET represent heat and humidity in the study area, which are two factors that are closely related to vegetation growth. The formula of GPP, LAI, FVC, LST, and WET is expressed as follows:

1. Gross primary productivity (GPP) and leaf area index (LAI)

GPP is an indicator of plant photosynthesis [40], which is often used to quantify the quality of vegetation growth [41]. Inversion of the LAI from MODIS data products has been verified by many scholars [42,43,44]. According to the product description of the LAI and GPP in MODIS data products, when the digital number (DN) value of the LAI is between 253 and 255, it represents bare land, snow area, or water area. For the valid DN value, the unit of the LAI is m^2^/m^2^, and the expression is as follows:

(1) $$\mathrm{LAI}=0.1\times {\mathrm{DN}}_{\mathrm{LAI}}$$

where ${\mathrm{DN}}_{\mathrm{LAI}}$ represents the DN value of leaf area index waveband images.

The effective value of the GPP band is between 0 and 30,000. For the effective DN value, the unit of GPP is g C m^−2^, and the calculation formula is:

(2) $$\mathrm{GPP}=0.1\times {\mathrm{DN}}_{\mathrm{GPP}}$$

2 Fractional vegetation coverage (FVC)

Many scholars have carried out several studies on vegetation coverage in ecologically fragile areas in China [45,46]. Satellite-derived NDVI data products are an indispensable choice in large-scale investigation and research [47,48]. Based on the MODIS product, the NDVI of Fuyun County was processed in this study, and FVC was estimated by pixel dichotomy. This method is used to calculate vegetation coverage based on the pixel linear decomposition model [49], and its expression is as follows:

(3) $$\mathrm{FVC}=\frac{\mathrm{NDVI}-{\mathrm{NDVI}}_{\mathrm{soil}}}{{\mathrm{NDVI}}_{\mathrm{veg}}-{\mathrm{NDVI}}_{\mathrm{soil}}}$$

The NDVI was selected as the parameter to calculate FVC. Since the measured data were difficult to obtain, the NDVI values of 95% and 5% of the confidence interval were used as the NDVI of the pure vegetation pixel and the pure soil pixel. The ${\mathrm{NDVI}}_{\mathrm{soil}}$ and ${\mathrm{NDVI}}_{\mathrm{veg}}$ values of 2008 were 0.0323 and 0.5124 in the study area, respectively. In 2018, the ${\mathrm{NDVI}}_{\mathrm{soil}}$ and ${\mathrm{NDVI}}_{\mathrm{veg}}$ values were 0.0827 and 0.6018 in the study area, respectively. The whole area was divided into three parts: when the NDVI was less than the NDVI of the pure soil pixel, the value of FVC was 0. When the NDVI was greater than the NDVI of the pure vegetation pixel, the value of FVC was 1. The FVC value of the pixel between the NDVI of the pure soil pixel and pure vegetation pixel was calculated by Equation (3).

3 Land surface temperature (LST) and modified wetness (WET)

Temperature and humidity are critical factors affecting vegetation growth and driving ecological environment changes. The DN value of LST_DAY_1 KM band in the MOD11 A2 data product was converted to the commonly used unit of degrees Celsius, and the formula is expressed as:

(4) $$\mathrm{LST}=0.02\times {\mathrm{DN}}_{\mathrm{LST}\_\mathrm{DAY}}-273.15$$

where ${\mathrm{DN}}_{\mathrm{LST}\_\mathrm{DAY}}$ denotes the DN value of the daytime land surface temperature waveband images.

Some indicators of humidity or dryness, such as the TVDI, are not suitable for measuring humidity due to the particularity of MBSs in arid regions. After comparison, it was found that the moisture component of tasseled cap transformation could reflect the comprehensive moisture of soil and vegetation in the arid area of NW China well, so it was used as the wetness indicator in this paper. The wetness indicator of the third component in the tasseled cap transformation reflects the moisture of soil, vegetation, and water. The tasseled cap transformation mainly exists in Landsat and IKONOS images, but some scholars had found that tasseled cap transformation also exists in MODIS images in recent years [50]. Based on the improved MODIS tasseled cap transformation formula and the surface reflectance product of MOD09A1 [51], the wetness indicator of Fuyun County was calculated by the following formula:

(5) $$\begin{array}{c} WET=0.1147b_{1}+0.2489b_{2}+0.2408b_{3}+0.3132b_{4}-0.3122b_{5}-0.6416b_{6}-0.5087b_{7}\# \end{array}$$

#### 2.3.2. The Calculation of the Ecological Quality Index

To avoid the influence of different units and data ranges of each indicator on the subsequent analysis, the five indicators were normalized, respectively, and the normalization formula is expressed as:

(6) $$N_{i}=\frac{\left(I-I_{\min}\right)}{I_{\max}-I_{\min}}$$

The five normalized indicators were recombined into a new image, and the indicator integration was carried out by using PCA. The research found that the covariance eigenvalue of the first principal component (PC1) was greater than 85% (Table 2); that is, PC1 could reflect the comprehensive information of the indicators, so it could be used to create the MODEI based on MODIS data.

(7) $$\mathrm{MODEI}=f\left(\mathrm{GPP},\mathrm{FVC},\mathrm{LAI},\mathrm{WET},\mathrm{LAT}\right)$$

The PC1 was selected as the initial MODEI. In order to facilitate the measurement and comparison of different years, the initial MODEI was standardized through Equation (8):

(8) $$\mathrm{MODEI}=\frac{\mathrm{PC}1-\mathrm{PC}1_{\min}}{\mathrm{PC}1_{\max}-\mathrm{PC}1_{\min}}$$

#### 2.3.3. Interpolation of Meteorological Data

Anusplin is special software for surface fitting of climate data based on spline interpolation theory, which has been widely used in studies worldwide [52,53]. The influence of DEM on meteorological factors is considered in the application [54,55]. Since there was only one national meteorological station in Fuyun County, the interpolation grid data surface of temperature and precipitation of the whole Xinjiang region was generated based on 54 national meteorological stations in Xinjiang, and then the data in the study area were extracted.

#### 2.3.4. Calculation of the Grazing Pressure Index

In order to analyze and evaluate the impact of grazing pressure on grassland, the pressure index of grazing was calculated in this study to quantify the grazing pressure [56]. The expression of the grazing pressure index (GPI) is as follows:

(9) $$\mathrm{GPI}=\frac{C_{s}}{C_{P}}$$

where GPI is the grazing pressure index of the grassland, $C_{s}$ is the actual carrying capacity (sheep units·ha^−1^), and $C_{P}$ is the theoretical carrying capacity (sheep units·ha^−1^). If GPI = 1, it indicates that the grassland is in the equilibrium state of herbage and livestock. If GPI > 1, the grassland has been overgrazed; if GPI < 1, it indicates that the grassland still has carrying capacity. The calculation methods of $C_{s}$ and $C_{P}$ are specified by Bi et al. [35].

#### 2.3.5. Spatial Auto-Correlation Analysis

Spatial auto-correlation is an important index reflecting the degree of correlation between a geographic phenomenon or an attribute value in a regional unit and the same phenomenon or attribute value in the adjacent regional unit. In this study, the classical global spatial auto-correlation was selected to test the spatial auto-correlation of the MODEI. When the absolute value of the global Moran’s I index is close to 1, it indicates stronger spatial auto-correlation. The formulas are expressed as:

(10) $$I=\frac{\sum_{i=1,j=1}^{n}W_{\mathrm{ij}}\left(x_{i}-\bar{x}\right)\left(x_{j}-\bar{x}\right)}{S^{2}\sum_{i=1,j=1}^{n}W_{\mathrm{ij}}}$$

(11) $$S^{2}=\frac{1}{n}\;\overset{n}{\underset{i=1}{\sum }}{\left(x_{i}-\bar{x}\right)}^{2}$$

where I represents the value of the global Moran’s I index; $S^{2}$ is the variance; $x_{i}$ and $x_{j}$ represent the attribute values of grid unit i and j, respectively; n is the number of grid cells;$\;\left(x_{i}-\bar{x}\right)$ is the deviation between the measured value and the mean value of grid cell i; $W_{\mathrm{ij}}$ is the normalized spatial weight matrix. The Moran’s I value is between −1 and 1. A value greater than 0 indicates positive auto-correlation, while a value less than 0 indicates negative auto-correlation.

Local spatial auto-correlation can measure the local spatial correlation degree and spatial differentiation between each grid and the surrounding grid [57]. In this study, the local Moran’s I is selected for spatial association or difference analysis of independent variables, characterized by the Moran scatter diagram and Lisa distribution diagram. It should be noted that the Lisa distribution must satisfy the Z test (α = 0.05), and the calculation formula of the local Moran’s I index is as follows:

(12) $$I_{i}=\frac{\left(x_{i}-\bar{x}\right)\sum_{j=1}^{n}W_{\mathrm{ij}}\left(x_{i}-\bar{x}\right)}{S^{2}}$$

(13) $$S^{2}=\frac{1}{n}\;\overset{n}{\underset{i=1}{\sum }}{\left(x_{i}-\bar{x}\right)}^{2}$$

## 3. Results

### 3.1. Basic Statistical Value of the Ecological Quality Index

The quantitative index is the crucial prerequisite for ecological quality evaluation. Therefore, the normalized indexes (GPP, FVC, WET, LST, and LAI) perform the integration of bands and then give the result of PCA as shown in Table 2. As can be seen from Table 2, the eigenvalue contribution rate of each index in the first principal component reached 85.5597% and 85.5477% in 2008 and 2018, respectively, which indicated that the first principal component had concentrated most of the variability information of the five indicators (Table 2). Therefore, the MODEI was formulated by integration of the five indicators via PC1. In the first principal component, FVC, GPP, LAI, and WET were all positive, indicating that they played a positive role in regulating the quality of the ecological environment. LST in PC1 was negative, indicating a negative impact on the quality of the ecological environment. The values of FVC and GPP in PC1 were higher than LAI, WET, and LST, indicating that FVC and GPP were the main influencing factors of ecological quality.

The mean values of the MODEI of Fuyun County in 2008 and 2018 were 0.292 and 0.303, respectively, with a gradually rising trend, indicating that the MODEI in the study area was improved. However, the mean values of the MODEI were all lower than 0.5, indicating that the MODEI in Fuyun County was generally low and increased at a low rate, which matched the characteristics in the arid area in NW China.

Among them, the mean values of FVC were 0.311 and 0.245 in 2008 and 2018, respectively, showing a decreasing trend with a small fluctuation. The mean value of GPP increased from 8.472 g C m^−2^ in 2008 to 11.231 g C m^−2^ in 2018, and the mean value of LAI increased from 0.303 m^2^/m^2^ in 2008 to 0.419 m^2^/m^2^ in 2018. The average WET in 2018 was higher, and the increases in WET and GPP were of great significance for the fragile arid area. The mean values of LST decreased from 33.716 °C in 2008 to 33.219 °C in 2018. The standard deviations of the five indicators were all large, indicating that the indicators in the study area have a significant degree of dispersion and strong spatial heterogeneity (Table 3). The decreasing trend of LST was coupled with the slight increase in GPP, LAI, and WET. Such effects of cooling, humidity, and greening improved the average MODEI in Fuyun County in the last ten years.

### 3.2. The Spatial Distribution of MODEI

To further explore the spatial distribution characteristics of the MODEI in 2008 and 2018, the MODEI was classified into five levels at intervals of 0.2 [23], including high (0.8–1), good (0.6–0.8), moderate (0.4–0.6), poor (0.2–0.4), and bad (0–0.2).

**Figure 2:**
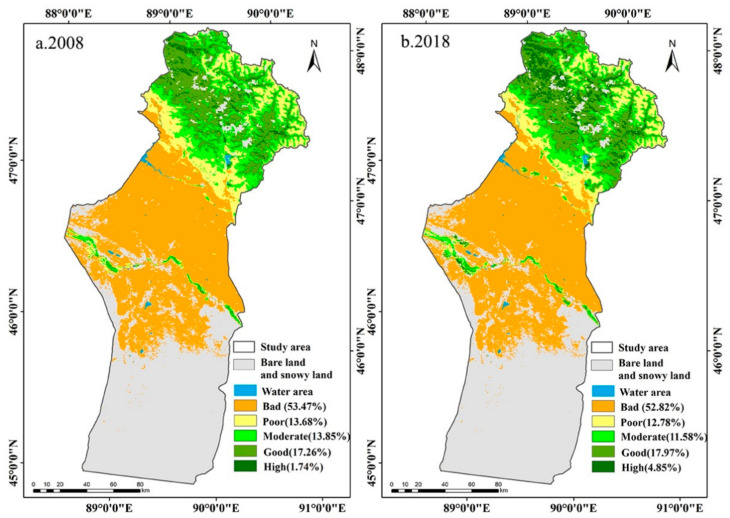
The spatial distribution of MODEI level in 2008 and 2018.

The ecological quality of Fuyun County presented a spatial pattern of “excellent in the north and poor in the south” (Figure 2). Among them, the areas of poor and bad levels were 16,208.56 km^2^ and 15,834.62 km^2^, accounting for 67.15% and 65.60% of the total area in 2008 and 2018, respectively (Table 4), indicating that most of the study area belonged to the ecologically fragile region. The area at the moderate level was 3343.37 km^2^ and 2795.152 km^2^, accounting for 13.85% and 11.58% of the total area. In 2008 and 2018, a total of 4586.898 km^2^ and 5509.227 km^2^ were at the good or high level of the MODEI, accounting for 19% and 22.82% of the study area (Table 4).

In terms of spatial distribution, the areas with excellent ecological quality were concentrated in the summer pastures at higher elevations and on both sides of the Irtysh River and the Ulungur River (Figure 2). The mean values of the MODEI in summer pastures were 0.59 and 0.62 in 2008 and 2018, respectively, showing an upward trend. However, the mean values of the MODEI of spring and autumn pastures and winter pastures were low and basically remained unchanged. In 2008, the mean values of the MODEI in spring and autumn pastures and winter pastures were 0.18 and 0.09, respectively; in 2018, the mean values of the MODEI in spring and autumn pastures and winter pastures were 0.19 and 0.09, respectively (Table 5).

**Figure 3:**
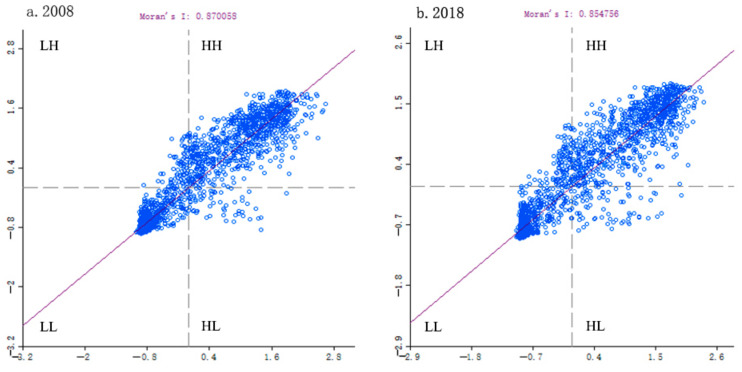
Moran scatter plots of MODEI in Fuyun County in 2008 and 2018. Note: HH: High–high cluster; LL: Low–low cluster; HL: High–low cluster; LH: Low–high cluster.

In order to better characterize the spatial differentiation characteristics of ecological environmental quality, a 3 km × 3 km grid was used to resample the spatial images of the MODEI in the study area in 2008 and 2018, and 2248 grids were obtained, respectively, which were used to further describe the spatial agglomeration characteristics of regional ecological quality. Figure 3 shows Moran scatter plots of the MODEI in Fuyun County in 2008 and 2018. The points in the four quadrants of the Moran’s I scatter plot, respectively, represent four different types of spatial connections formed between a certain area and the surrounding area. The grids were mainly distributed in the first quadrant and the third quadrant, indicating that the distribution of the MODEI had an obvious spatial positive correlation, showing a clear trend of high–high (HH) and low–low (LL) aggregation, and the clustering effect of grids and adjacent grids was significant. The degree of auto-correlation in 2018 was relatively weak compared with that in 2008, implying that HH aggregation or LL aggregation in the MODEI was slightly weakened in 2018, while the random distribution trend was slightly enhanced.

The point clustering in the first quadrant indicated that the MODEI values in the areas with higher ecological environment quality had few differences, and the same situation happened in the areas with lower ecological environment quality, as shown by the point clustering in the third quadrant. In addition, the Monte Carlo simulation method was used to test the significance of Moran’s I in Geoda095i, and the *p* values of both phases were equal to 0.001, suggesting that the spatial auto-correlation was significant at a 99.9% confidence level.

**Figure 4:**
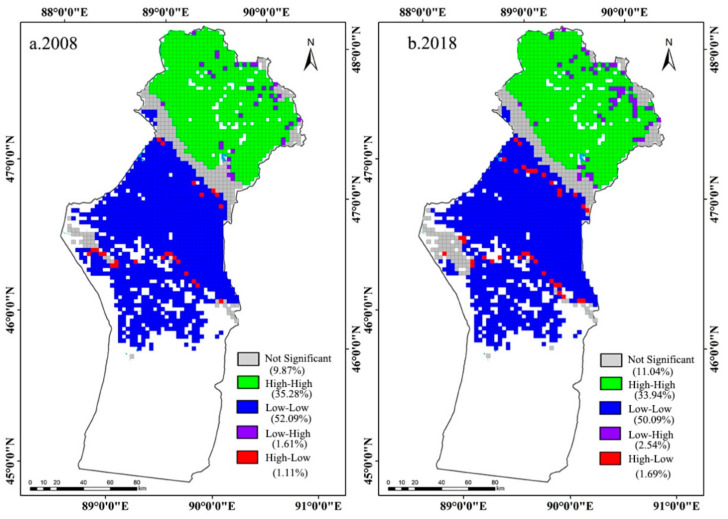
LISA aggregation map of MODEI in Fuyun County.

**Figure 5:**
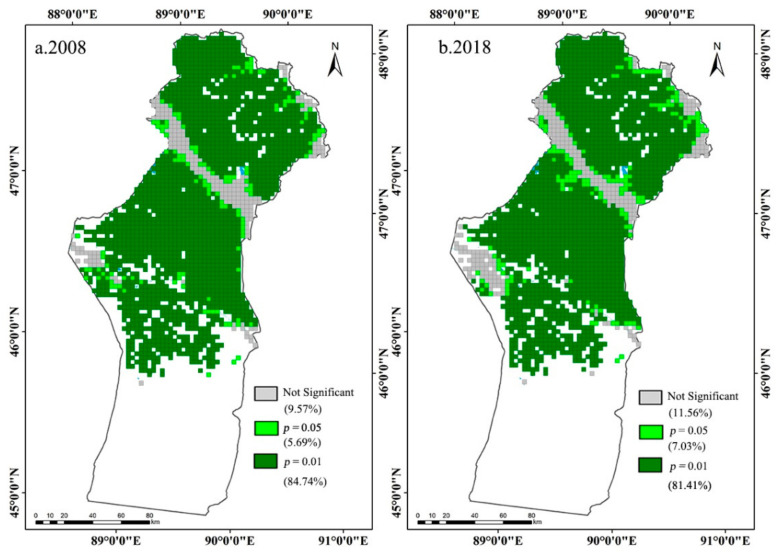
LISA significance map of MODEI in Fuyun County.

The spatial distribution features of the MODEI were visualized by the LISA clustering map and the LISA significance level map (Figure 4 and Figure 5). As shown in Figure 4, the HH area was mainly distributed in summer pastures, while the LL area was located primarily in spring and autumn pastures and winter pastures. The LH region was scattered in the HH region of the summer pastures. The HL region was mainly distributed along the Irtysh River and the Ulungur River. Compared with 2008, the LH area and HL area expanded in 2018. A percentage of 84.74 and 81.41 of the region reached the significance level of 0.01 in 2008 and 2018, respectively. The region of the significance level of 0.05 was mainly distributed at the edge of the 0.01 significance area and the edge of the two rivers (Figure 5).

### 3.3. Changes in MODEI from 2008 to 2018

**Figure 6:**
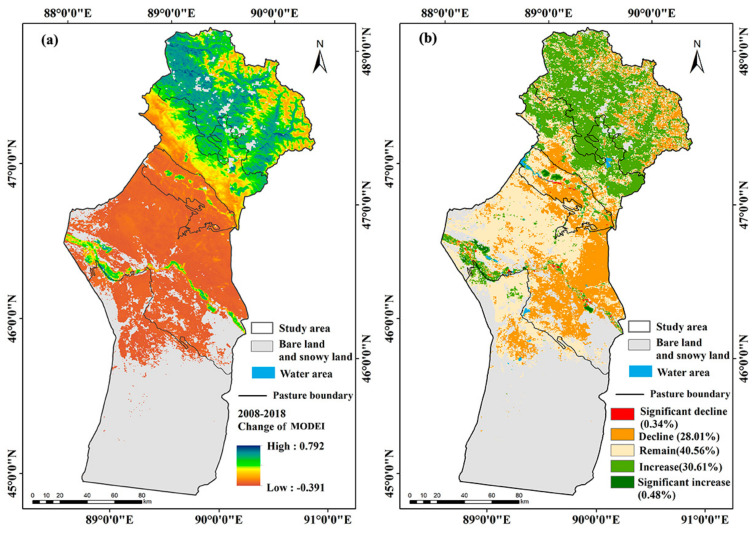
Changes in MODEI from 2008–2018 in Fuyun County. (**a**) 2008; (**b**) 2018.

Figure 6a illustrates the dynamic changes of the MODEI in Fuyun County during the decade. The variation range of the MODEI in the study area was between −0.391 and 0.792. Combined with the natural breakpoint method in ArcGIS, the changed values of the MODEI were divided into five intervals: significant decline (−0.391 to −0.1), decline (−0.1 to −0.015), remained unchanged (−0.015 to 0.015), increase (0.015 to 0.39), and significant increase (0.39 to 0.792). Based on this, the spatial distribution map of the dynamic change of the MODEI in the study area was obtained (Figure 6b). Table 6 further provided statistics on the detailed changes of pastures in different seasons of the whole region.

Statistical analysis showed that the region with improved ecological quality was 7511.42 km^2^, accounting for 31.12% of the study area. The significantly improved areas accounted for 0.47% of the whole area. Among them, 4587.03 km^2^ was distributed in summer pastures and 2552.33 km^2^ and 372.06 km^2^ were distributed in spring and autumn pastures and winter pastures, accounting for 19.01%, 10.57%, and 1.54% of the study area, respectively. The area where the MODEI was unchanged was 9788.42 km^2^, accounting for 40.56% of the study area, with the largest proportion (Table 6).

The region where the ecological quality deteriorated was 6838.16 km^2^ and accounted for 28.32% of the total area. Within this area, 1307.08 km^2^ was distributed in summer pastures and 4225.22 km^2^ and 1305.86 km^2^ were distributed in spring and autumn pastures and winter pastures, occupying 5.41%, 17.5%, and 5.41% of the study area, respectively (Table 6). The areas where the MODEI declined were mainly located in the high-altitude mountains in the northernmost part of summer pastures and spring and autumn pastures and winter pastures. The places where the MODEI decreased significantly were situated on both sides of the river (Figure 6).

**Figure 7:**
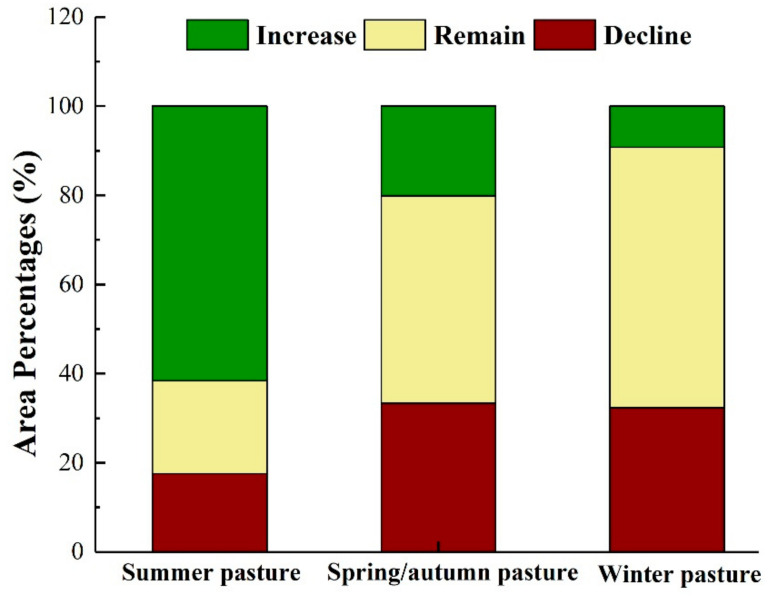
Probability statistics of variation of MODEI in different seasonal pastures.

In addition, the changes in the MODEI in different seasonal pastures in the study area from 2008 to 2018 were statistically analyzed (Figure 7). The statistical results showed that the ecological quality of most summer pasture areas was improved, and the areas where the ecological environment deteriorated were mainly located in spring and autumn pastures and winter pastures. The area where the MODEI decreased accounted for 15% of the summer pasture area, 20.81% and 61.73% of the summer pasture remained unchanged and increased in the MODEI. In spring and autumn pastures, the areas where the MODEI decreased occupied 33.37%, those that remained unchanged occupied 46.47%, and those where it increased occupied 20.16%. Among winter pastures, 32.38% of the area’s MODEI became worse, 58.39% of the region remained unchanged, and in 9.23% it increased.

### 3.4. Simulation and Prediction of the MODEI

Taking the MODEI as the dependent variable and GPP, FVC, WET, LAI, and LST as the independent variables, 2248 samples were collected in 2008 and 2018. The simulation and prediction models of the MODEI of Fuyun County in 2008 and 2018 were established. The regression models were as follows (significant at the 0.01 level):

(14) $${\mathrm{MODEI}}_{2008}=0.265\mathrm{GPP}+0.447\mathrm{FVC}+0.133\mathrm{WET}+0.170\mathrm{LAI}-0.231\mathrm{LST}+0.109\;\left(R^{2}=1\right)$$

(15) $${\mathrm{MODEI}}_{2018}=0.271\mathrm{GPP}+0.473\mathrm{FVC}+0.127\mathrm{WET}+0.150\mathrm{LAI}-0.242\mathrm{LST}+0.150\;\left(R^{2}=1\right)$$

From the absolute value of regression coefficients of each index, FVC had the most significant impact on ecological quality in Fuyun County, followed by GPP, LST, LAI, and WET, which were consistent with the results of principal component analysis.

**Figure 8:**
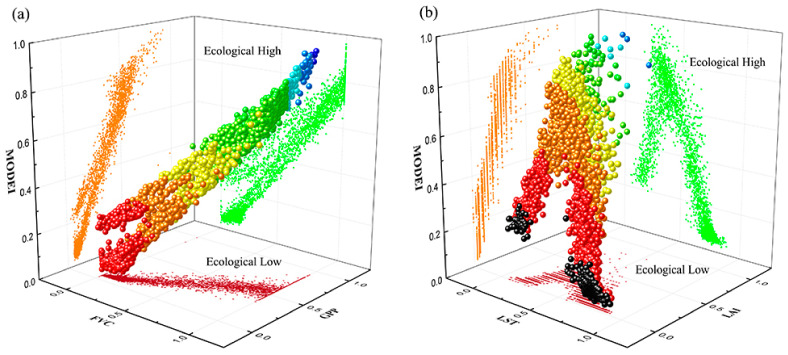
(**a**): Three-dimensional scatter plots illustrating the relationships among MODEI, FVC and GPP; (**b**): Three-dimensional scatter plots illustrating the relationships among MODEI, LST and LAI.

In addition, a total of 4496 points were collected in 2008 and 2018. The relationship between the MODEI and the four indicators with the largest contribution rate (FVC, GPP, LST and LAI) was analyzed, and the MODEI samples were projected into the three-dimensional space (Figure 8). In Figure 8a, the top of the scatter plot represents the area with better ecological quality, which has higher FVC and higher GPP. Conversely, the bottom of the scatter plot represents the area with poor ecological quality, which has lower FVC and lower GPP. Multiple linear regression models also showed that both FVC and GPP had a positive contribution to ecological quality. The top of the scatter plot represents an excellent ecological environment with higher LAI and lower LST (Figure 8b), indicating that LST and WET had opposite effects on ecological quality, which was consistent with the results of principal component analysis and regression models.

## 4. Discussion

### 4.1. Climate Change and Its Influence on the MODEI

Ecosystems are sensitive to climate change, especially in arid areas [58]. Climate change can directly affect vegetation growth in the ecosystem through precipitation and temperature by affecting ground humidity, vegetation productivity, etc. [59]. Our results showed that the indicators of FVC, GPP, LAI, and WET had positive effects on the MODEI, while the LST indicator had a negative impact on the MODEI, results which were consistent with previous studies [29,60].

**Figure 9:**
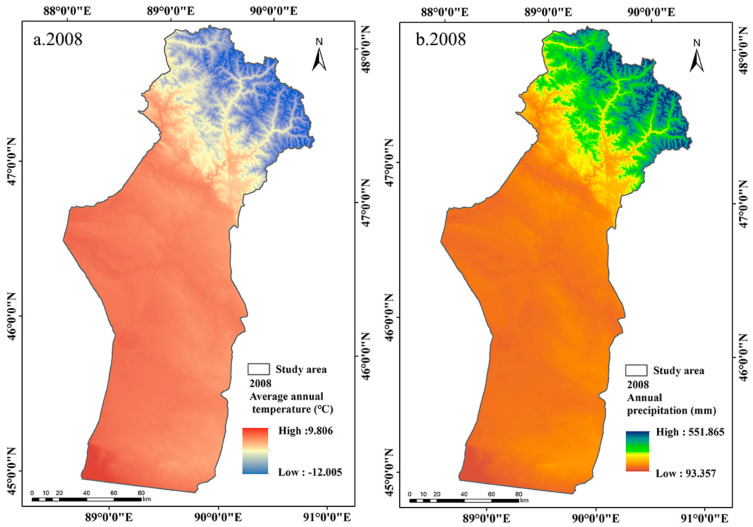
Spatial distribution of average temperature and annual precipitation in 2008.

**Figure 10:**
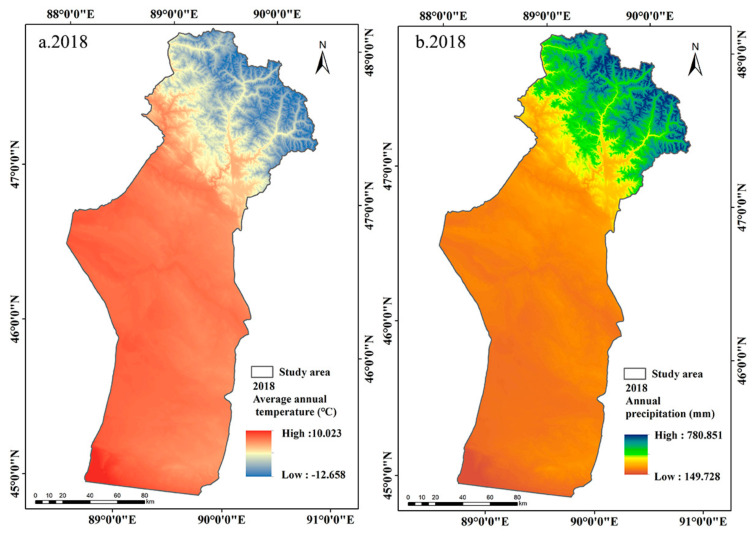
Spatial distribution of average temperature and annual precipitation in 2018.

The annual precipitation in Fuyun County showed a fluctuating upward trend from 2008 to 2018 (Figure 9b and Figure 10b). The annual precipitation in the study area increased from 185.01 mm in 2008 to 289.48 mm in 2018, with an average increase of 10.45 mm/year (Figure 9 and Figure 10). The study carried out by Liang et al. [61] showed that the vegetation productivity in arid and semi-arid areas was mainly controlled by precipitation. Zhang and Ren [62] proved that meteoric precipitation was the main source of vegetation moisture in arid ecosystems in Central Asia, which can increase the soil water content, so the soil can provide more water for the vegetation to enhance its photosynthesis, thus improving the growth of the vegetation [63]. Therefore, the improvement of ecological conditions in the study area was mainly driven by the increase in precipitation, as the increase in precipitation directly increases humidity thus improves vegetation growth. The results showed that the decreased MODEI of summer pastures was mainly concentrated in the high-altitude mountainous area with abundant precipitation in the northernmost summer pastures (Figure 6b). However, when the precipitation exceeds the water level required for vegetation growth, the photosynthesis of vegetation will be inhibited due to the limited solar radiation and the increased relative humidity [35,64].

**Figure 11:**
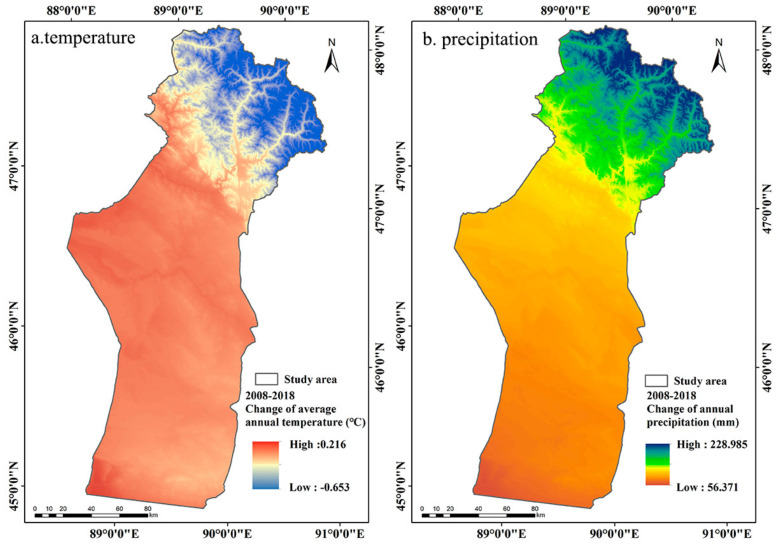
Spatial distribution map of the changes in average temperature and annual precipitation from 2008 to 2018.

Previous studies have shown that most vegetation in arid regions of Central Asia was more sensitive to changes in precipitation than to changes in temperature [33,65]. The average temperature in the study area in 2008 and 2018 was 3.73 °C and 3.74 °C, respectively, with no noticeable trend of change (Figure 9a and Figure 10a). The areas where the temperature dropped were mainly located in summer pastures, while spring and autumn pastures and winter pastures appeared to have a slight warming trend (Figure 11a). PCA and prediction model results showed that LST had a negative effect on the MODEI (Table 2). Therefore, in addition to the influence of precipitation change and human factors, the decreased temperature in summer pastures and the increased temperature in spring and autumn pastures and winter pastures may play a part in the increase in the MODEI in summer pastures and the reduction in the MODEI in spring and autumn pastures and winter pastures.

### 4.2. Impact of Human Activities on the MODEI

#### 4.2.1. Changes in the Grazing Pressure Index

For Fuyun County, grassland is the primary ecosystem type in this region, and grazing is the most basic utilization mode of grassland. It is also a major factor among human activities disturbing grassland [66]. Therefore, our study analyzed the grazing pressure index of grassland from 2001 to 2018.

**Figure 12:**
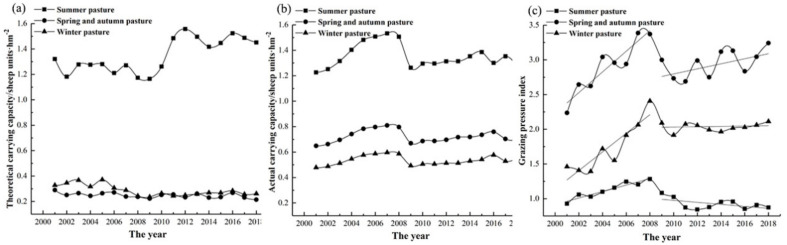
Changes in theoretical carrying capacity (**a**), actual carrying capacity (**b**), and grazing pressure index (**c**) in Fuyun County from 2001 to 2018.

From 2001 to 2008, the average values of the GPI in summer pasture, spring and autumn pasture, and winter pasture were 1.13, 2.90, and 1.74 from 2001 to 2008 and 0.93, 2.95, and 2.03 between 2009 and 2018, respectively. The GPI of summer pastures, spring and autumn pastures, and winter pastures showed an upward trend from 2001 to 2008 and reached the maximum in 2008 (Figure 12). As shown in Figure 12c, since 2008, the grassland GPI of summer pastures has shown a fluctuating downward trend. Therefore, the improvement of the MODEI in summer grassland may be related to the fluctuating decrease in the GPI after 2008. In addition, the improvement of the MODEI in summer pasture was possibly related to the grassland ecological protection engineering measures implemented in recent years [67]. From 2009–2018, the slopes of the GPI of spring and autumn pastures and winter pastures were positive, showing an increasing trend, especially in spring and autumn pastures. Therefore, the rising grazing pressure may be the main reason for the deterioration of the ecological quality in spring and autumn pastures and winter pastures. Chen et al. [66] also pointed out that grazing was the main driving force of grassland changes caused by human activities.

The statistical results in Table 6 show that the area where the MODEI of spring and autumn pastures declined reached 4225.22 km^2^, and the area where the MODEI of winter pastures decreased was 1305.86 km^2^. The area of MODEI decline of winter pasture was much smaller than that of spring and autumn pasture. The reasons are as follows: firstly, the utilization period of spring and autumn pastures is the crucial period for forage germination in spring and seed set in autumn, and the excessive utilization intensity affects the growth and reproduction of herbage. Secondly, Figure 12c shows that although the GPI of winter pasture showed an upward trend from 2009 to 2018, the trend was not apparent. In addition, a survey of herders showed that after 2010, the number of herders grazing in winter decreased, and most herders implemented captive breeding in winter [38]. This measure also controlled the deterioration of the ecological quality of winter pastures.

#### 4.2.2. Changes in Land Use

Severe human disturbances (such as higher population densities and the expansion of construction land) can put enormous pressure on ecosystems, resulting in a decline in ecological quality [68,69].

The areas where the MODEI had significantly decreased were concentrated on both sides of the Irtysh River and the Ulungur River. Since 2006, the government has set up new settlement villages with unified housing standards along the banks of the Ulungur River to encourage the herders to settle down, improve their living conditions and relieve the pressure on the grasslands. The resettlement project for herders began on a large scale in 2009 [38]. Due to the implementation of the herdsmen settlement project, the area of construction land increased. The alluvial plain of the Ulungur River has relatively flat terrain and superior natural conditions, so the construction of newly settled villages was mainly concentrated on Ulungur River banks. According to statistics of land use change data, the area of land for industrial and mining residents was 16.89 km^2^ and 71.94 km^2^ in 2008 and 2018, respectively, increasing by 3.26 times. Therefore, due to the expansion of construction land, some areas on both sides of the river have shown a downward trend in the MODEI in the past ten years.

**Figure 13:**
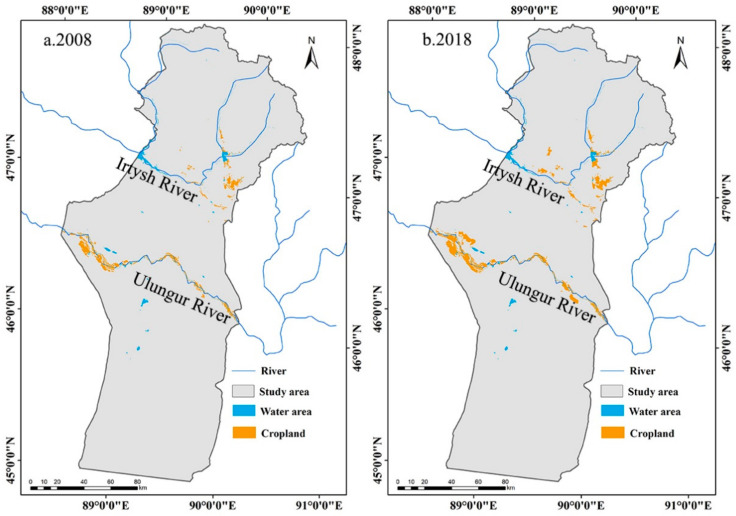
Spatial distribution map of cropland in 2008 and 2018.

In addition, the MODEI improved significantly in the areas that were also distributed on both sides of the Irtysh and Ulungur rivers, which was possibly caused by the settlement project of herdsmen. The families of herdsmen who moved to the newly settled village were given an average of 3.3 hectares of farmland to plant artificial grassland [38]; as a result, the area of artificial grassland has significantly increased. In addition to artificial grassland, the area of cropland also increased. The cropland area in Fuyun County was 378.39 km^2^ and 474.38 km^2^ in 2008 and 2018, respectively, with an increase of 25.37% (Figure 13). Therefore, due to the planting of artificial grassland and the increase in cropland, the MODEI of some areas on both sides of the river improved.

### 4.3. Limitations and Future Perspectives

The improved ecological quality index in this paper is a relatively reliable and effective ecological quality assessment method [70]. Its evaluation results can provide valuable scientific references for ecological management. However, there are still some limitations that need to be improved in future studies. For example, evaluating ecological quality should be more comprehensive, and more indicators such as biodiversity index, water density index, land use intensity, and building land index should be considered in future studies.

Despite the above limitations, the method of the MODEI adopted in this study is not complicated in operation and can be used to evaluate ecological quality quickly and effectively. In future studies, we will strive to improve the accuracy of the MODEI model for to be more widely used and promoted. Firstly, some indicators reflecting the biodiversity and land fragility index will be added to the ecosystem assessment system of the mountain–basin systems in arid regions. Secondly, the potential driving factors affecting the regional ecological quality will be further discussed to provide a scientific basis for sustainable utilization and effective management of mountain–basin ecosystems in arid areas.

## 5. Conclusions

This paper used MODIS data to extract the GPP, FVC, LAI, LST, and WET information, and a MODEI model was established to analyze and evaluate the ecological environmental quality of Fuyun County and the spatial differentiation characteristics of MODEI were discussed. The research found that: (1) FVC, GPP, LAI, and WET had a positive impact on the MODEI, while LST negatively impacted the MODEI; among them, FVC and GPP had a more significant effect on the quality of the ecological environment. (2) The average values of the MODEI in the study area in 2008 and 2018 were 0.292 and 0.303, respectively, indicating that the MODEI in the study area was developing towards a better trend. The MODEI in Fuyun County showed a spatial pattern of “excellent in the north and poor in the south”. The areas of poor and bad levels accounted for 67.15% and 65.60% of the total area in 2008 and 2018, respectively. The areas of a moderate level accounted for 13.85% and 11.58% of the total area in 2008 and 2018, respectively. In 2008 and 2018, the areas of the good and high levels of MODEI accounted for 19% and 22.82% of the study area in 2008 and 2018, respectively. The values of Moran’s I were high in both periods, which indicated that the spatial distribution was clustered rather than random. (3) The improvement of the ecological conditions in the study area was mainly driven by the increase in precipitation. The areas where the MODEI increased were mainly located in the summer pastures, which was ascribed to the decrease in the GPI and increased precipitation in the summer pastures. The areas where the MODEI increased significantly were located along the river, which was mainly caused by the planting of artificial grassland along the river. The areas where the MODEI was basically unchanged were primarily distributed in spring and autumn pastures and winter pastures. The areas where the MODEI decreased were mainly distributed in the northern part of summer pastures situated in high-altitude mountains, and the spring and autumn pastures and winter pastures, and the areas where the MODEI decreased significantly were mainly located on both sides of the rivers. To a certain extent, the increase in the GPI could explain the deterioration of the ecological quality of the spring and autumn pastures and winter pastures. In addition, due to the expansion of urban and rural residential land, the ecological quality of some areas on both sides of the river has seriously deteriorated.

In view of the deterioration of the ecological quality of spring and autumn pastures and winter pastures, measures need to be taken to reduce the intensity of grazing. Firstly, the local government can continue to implement the herdsmen settlement project and increase the diversity of herdsmen’s livelihoods, and the intensity of grassland grazing can be reduced through captive breeding and corresponding infrastructure. Secondly, it is necessary to carry out the planting of stable and high-yield, high-quality forage grass. Combined with the natural conditions of the study area, large-scale construction and planting of high-quality and high-yield artificial forage grass should be carried out along the Irtysh River near the spring and autumn pasture and the Ulungur River near the winter pasture to provide stable and sufficient forage for livestock and achieve the purpose of using a small area of high-quality and high-yielding artificial grassland in exchange for the protection and restoration of a large area of natural grassland. Planting artificial grassland can solve the problem of extended use time of spring and autumn pastures and the wide difference between the grassland area and the carrying capacity of winter pastures, and reduce the pressure on the grasslands. These suggestions can provide a scientific basis for ecological restoration and ecological quality improvement of mountain–basin systems in arid areas and other similar ecosystems throughout the world.

## Figures and Tables

**Table 1:** Research indicators and MODIS data products.

Indicator	Product	Extracted Band	Temporal Resolution (d)	Spatial Resolution (m)
GPP	MOD17A2H	Gpp_500 m	8	500
FVC	MOD13A1	500m_16_days_NDVI	16	500
LAI	MOD15A2H	Lai_500 m	8	500
WET	MOD09A1	sur_refl_b01 to 07	8	500
LST	MOD11A2	LST_Day_1 km	8	1000

Note: GPP: gross primary productivity; FVC: fractional vegetation coverage; LAI: leaf area index; WET: wetness; LST: land surface temperature.

**Table 2:** Principal component eigenvalues.

Year	Indicators	PC1	PC2	PC3	PC4	PC5
2008	FVC	0.73481	0.34362	0.02455	0.57563	−0.10015
	GPP	0.43585	0.20132	−0.03644	−0.55731	0.67645
	LAI	0.27947	0.1872	0.07268	−0.59759	−0.72419
	LST	−0.37923	0.74486	0.54284	0.02957	0.07628
	WET	0.21949	−0.50153	0.83553	−0.00846	0.04589
	Eigenvalue	0.04673	0.00551	0.00177	0.00048	0.00013
	Percent eigenvalue	85.5597	10.0876	3.2419	0.8798	0.231
2018	FVC	0.75333	0.3866	0.09882	−0.52096	−0.04318
	GPP	0.43101	0.15924	−0.04565	0.68619	0.56208
	LAI	0.23866	0.10684	0.05217	0.50247	−0.82245
	LST	−0.38577	0.70148	0.59216	0.07011	0.05958
	WET	0.20235	−0.56718	0.79673	0.01892	0.04714
	Eigenvalue	0.0543	0.00737	0.00115	0.0005	0.00016
	Percent eigenvalue	85.5477	11.606	1.8116	0.7863	0.2484

**Table 3:** MODEI and related indicator values of 2008 and 2018.

Indicator	2008				2018			
	Minimum	Maximum	Mean	Standard Deviation	Minimum	Maximum	Mean	Standard Deviation
GPP	0	36.4	8.472	6.863	0	45.6	11.231	9.007
FVC	0	1	0.311	0.288	0	1	0.245	0.313
LAI	0	2.2	0.303	0.274	0	3.7	0.419	0.414
WET	0.005	0.582	0.231	0.081	0.006	0.68	0.255	0.093
LST	6.17	47.13	33.716	8.251	6.769	47.01	33.219	8.516
MODEI	0	1	0.292	0.254	0	1	0.303	0.278

Note: GPP: gross primary productivity; FVC: fractional vegetation coverage; LAI: leaf area index; WET: wetness; LST: land surface temperature; MODEI: MODIS data-based ecological index.

**Table 4:** Area and proportion of MODEI level in 2008 and 2018.

Level	2008		2018	
	Area (km^2^)	%	Area (km^2^)	%
Bad (0–0.2)	12906.14	53.47	12750.58	52.82
Poor (0.2–0.4)	3302.422	13.68	3084.042	12.78
Moderate (0.4–0.6)	3343.37	13.85	2795.152	11.58
Good (0.6–0.8)	4166.77	17.26	4338.041	17.97
High (0.8–1.0)	420.1287	1.74	1171.186	4.85

**Table 5:** MODEI of different seasonal pastures.

Pasture Type	2008			2018		
	Minimum	Maximum	Mean	Minimum	Maximum	Mean
Spring and autumn pasture	0.03	0.95	0.18 ± 0.17	0.01	0.96	0.19 ± 0.19
Winter pasture	0	0.76	0.09 ± 0.05	0	0.89	0.09 ± 0.08
Summer pasture	0.2	1	0.59 ± 0.16	0.19	1	0.62 ± 0.18

**Table 6:** Changes in MODEI in Fuyun County between 2008 and 2018.

Pasture Type	Change in *p* Values		Area (km^2^)	Percentage (%)	Total Percentage (%)
Summer pasture	Decline	Significant decline	17.72	0.07	5.41
		Decline	1289.36	5.34	
	Remain	Remain	1548.52	6.42	6.42
	Increase	Increase	4587.03	19.01	19.01
		Significant increase	--	--	
Spring and autumn pasture	Decline	Significant decline	41.69	0.17	17.5
		Decline	4183.53	17.33	
	Remain	Remain	5884.9	24.38	24.38
	Increase	Increase	2476.77	10.26	10.57
		Significant increase	75.56	0.31	
Winter pasture	Decline	Significant decline	23.62	0.1	5.41
		Decline	1282.24	5.31	
	Remain	Remain	2355	9.76	9.76
	Increase	Increase	333.33	1.38	1.54
		Significant increase	38.73	0.16	
Whole region	Decline	Significant decline	83.03	0.34	28.32
		Decline	6755.13	27.98	
	Remain	Remain	9788.42	40.56	40.56
	Increase	Increase	7397.13	30.65	31.12
		Significant increase	114.29	0.47	

## Data Availability

All data generated or analyzed during this study are included in this published article.
